# Evidence-Based Medicine and Good Clinical Practice in Research in Pediatric and Adolescent Medicine

**DOI:** 10.3390/children12101309

**Published:** 2025-09-29

**Authors:** Ageliki A. Karatza, Asimina Tsintoni, Dimitrios Kapnisis, Despoina Gkentzi, Sotirios Fouzas, Eirini Kostopoulou, Xenophon Sinopidis, Nikolaos Antonakopoulos

**Affiliations:** 1 Department of Pediatrics, University of Patras Medical School, 26504 Patras, Greece; karatza@upatras.gr (A.A.K.); a.tsintoni@upatras.gr (A.T.); gkentzid@upatras.gr (D.G.); sfouzas@upatras.gr (S.F.); irekost@upatras.gr (E.K.); xsinopid@upatras.gr (X.S.); 2 Division of Fetal–Maternal Medicine, Department of Obstetrics and Gynecology, University of Patras Medical School, 26504 Patras, Greece; nantonakop@upatras.gr

**Keywords:** evidence-based, medicine, clinical, practice, pediatrics

## Abstract

**Highlights:**

**What are the main findings?**

- Evidence-Based Medicine is increasingly valued and popular in modern medical societies, and its practice involves a systematic process to integrate robust medical literature into daily practice.
- Adherence to Good Clinical Practice is crucial as it facilitates the generation of high-quality studies that form the reliable evidence used in EBM.

**What is the implication of the main finding?**

- Help physicians integrate the best available evidence to care for young patients more efficiently.
- Apply the basic good practice principles for conducting trials in children and adolescents.

**Abstract:**

Practicing medical research based on the best evidence is gaining increased value and popularity among most medical societies in the current era. Good clinical practice (GCP) is internationally recognized as the scientific and ethical standard for the design, conduct, performance, auditing, recording, analysis, and reporting of clinical trials involving human subjects. GCP ensures the accuracy and credibility of trial while safeguarding the rights, integrity, and confidentiality of participants. Adherence to GCP facilitates the generation of high-quality studies that can be incorporated in Evidence-Based Medicine (EBM). The clinical practice of EBM seeks to integrate robust medical literature into daily medical practice. This process involves systematically searching for high-quality evidence, critically appraising the retrieved literature, applying sound clinical principles and finally evaluating the efficacy of the chosen approach. Although EBM has been evaluated in many resource settings, it has not been addressed sufficiently in the field of Pediatrics and more specifically in indigenous populations. In this review, we briefly explain the EBM approach and its applications in Pediatrics, in order to help physicians care for young subjects more efficiently by integrating the best available information into their routine clinical practice. Also, the basic good practice principles for conducting clinical trials in children and adolescents are highlighted, emphasizing the importance of applying high ethical principles in this vulnerable population.

## 1. Introduction

Evidence-based medicine (EBM) involves using the best available evidence to guide decisions on diagnosis, treatment, and prevention strategies, while tailoring these approaches to the specific characteristics and circumstances of individual patients or populations, as well as the resources available to healthcare providers [1,2]. The past decade has seen substantial progress, resulting in a wealth of information now accessible for many healthcare decisions. Physicians and patients can currently take advantage of the best available evidence by identifying the most reliable resources pertinent to their specific clinical concerns [1].

Integrating EBM into medical decision-making is an ongoing and adaptive process designed to improve the efficiency and quality of clinical practice, particularly for busy pediatricians [3]. Several studies reveal that Pediatricians perceive the lack of time, knowledge, and resources as barriers to implementing EBM in clinical settings. Recently, there have been developments to help Pediatricians overcome these barriers and use EBM to identify access, apply, and integrate new knowledge to provide high-quality care for their young patients [3]. However, an insufficient number of publications include patient-oriented outcomes in Pediatrics and specific pediatric populations. Staying up to date with the exponentially increasing quantity of published evidence is not an easily accomplished task for healthcare providers [3]. EBM should minimize the interval between the dissemination of clinically relevant evidence and its integration into general practice, increase the number of patients being treated with the best up-to-date therapy, allowing Pediatric practice to ameliorate with time [4].

Efficient literature searching and the application of formal rules of evidence in evaluating clinical literature are necessary for EBM. Knowledge translation is EBM’s future challenge, which ensures that day-to-day decision-making is guided by sound principles and the most up-to-date evidence. The principles of EBM have become core concepts of all stages of continuing medical education, as well as courses, workshops, and online resources. Clinical expertise has now become the ability to incorporate research evidence and patients’ circumstances to help patients make optimal decisions. Even more challenging is ensuring decisions are consistent with the patients’ values. The widespread and appropriate application of EBM across all clinical practices remains an aspirational goal. While significant progress has been achieved, persistent challenges suggest that the next decade of EBM development will be as dynamic and transformative as the first [5].

**Figure 1:**
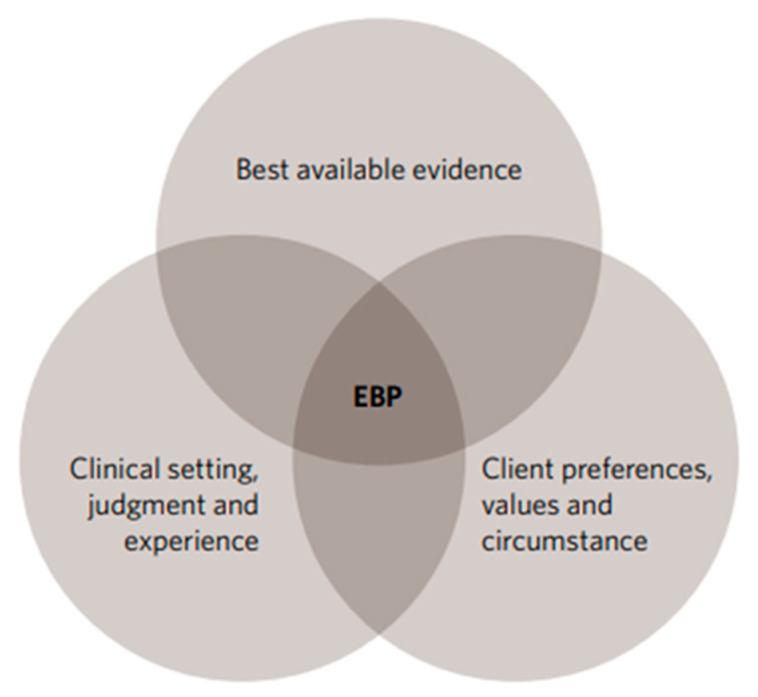
Components of evidence-based practice. In: A quick reference guide to evidence translation. What are sources of evidence? Reproduced with permission from © Orygen [6].

As a Pediatrician attempts to incorporate EBM into routine clinical practice specific barriers must be acknowledged: (1) limited time, (2) insufficient skills and knowledge, (3) restricted access to information technology and resources, (4) challenges in translating best evidence into clinical decisions, (5) perceived loss of physician autonomy, (6) patient expectations discord with empiric evidence, and (7) financial constrains [3]. After acknowledging these barriers in applying EBM in Pediatrics, determining the best way to incorporate it into patient care has been facilitated (Figure 1) [2]. This approach helps pediatricians stay up to date with medical knowledge and provides high-quality care [1].

## 2. The Definition of EBM

The term evidence-based medicine (EBM) and the first concise description of its characteristics were described about 25 years ago and were defined by G. Guyatt [2,7]. This term consists of a systematic search of the literature to identify best available evidence, critical appraisal of the retrieved literature, applying the results in medical practice, and finally evaluating the efficacy of the approach. There is a standard method used in EBM practice for searching literature and appraising existing evidence [2,7]. This process has been shown to significantly reduce the time required to incorporate information into daily practice [3]. Although EBM was first described in 1991 by Guyatt [7] the first randomized clinical trial was performed by James Lind in 1747. He was a surgeon in the Royal Navy who conducted a simple randomized controlled trial. He demonstrated that sailors with scurvy dramatically improved their symptoms when they added citrus to their diet compared with other treatments. In spite of the fact that the results were quite compelling, it took years for the study to be published and citrus fruits to become a principal part of sailors’ diets [8].

Application of the following five main steps helps to systematically organize and utilize current data into clinical practice, as shown in Table 1. In this way, the final outcomes are more reproducible compared to the traditional medical practice, which represents the primary distinction between these two approaches [9].

## 3. The PICO Model: Structure a Clinical Question

Asking an “answerable” clinical question about a specific patient setting is the first step of EBM in clinical practice. Special attention should be paid to the construction of the question; otherwise, the further steps will not be successful [10]. The PICO model is the most widely used model for formulating appropriate and effective clinical questions. Since its introduction, it has served as a significant conceptual model in EBM [11]. First, it engages the clinician to focus on the patient’s belief of the most important concern. Second, it facilitates computerized literature search by guiding the questioner to select appropriate keywords or language parameters. Third, it directs the user to identify clearly the problem, the required intervention, and expected outcomes to ensure optimal care for a specific patient. The Cochrane Handbook for Systematic Reviews of Interventions recommends using the PICO framework to formulate review questions, thereby ensuring that all relevant components of the question are clearly defined [12].

**PICO** represents the first letter of the words: **P**atient, **I**ntervention, **C**omparison, **O**utcome.

The question in this model is outlined by defining [10]:

- **(P)atients** or populations which may belong to a specific group (refugees, immigrants, Roma, single-parent families, unemployed parents).
- **(I)ntervention** refers to the kind of treatment or procedure whose effectiveness we aim to evaluate.
- **(C)omparison** refers to the standard treatment or alternative option used as a benchmark to assess the effect of the intervention.
- **(O)utcome** meaning the intended outcome we expect from our intervention.

## 4. Levels of Organization of EBM: “Summaries”, “Synopses”, “Syntheses”, “Studies” and “Systems” and the “Pyramid” Models in EBM

**Figure 2:**
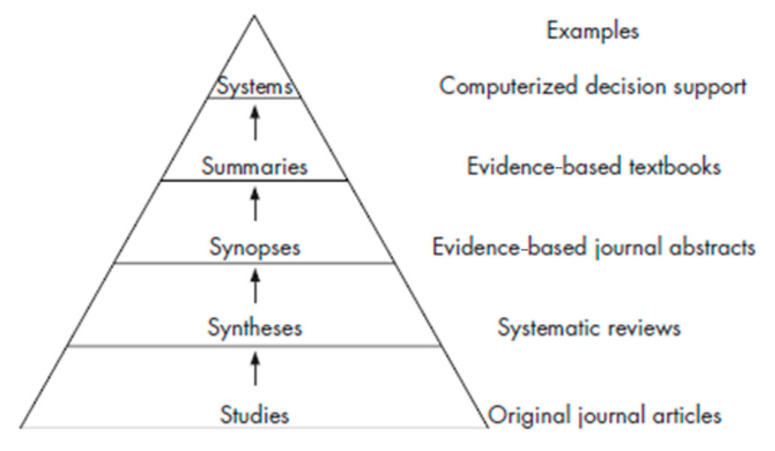
The (5S) levels of organization of EBM for healthcare decisions. EBM notebook. Of studies, syntheses, synopses, summaries, and systems: the ‘‘5S’’evolution of information services for evidence-based healthcare decisions. EBM Volume 11 December 2006. (with permission from the publisher) [15].

**Figure 3:**
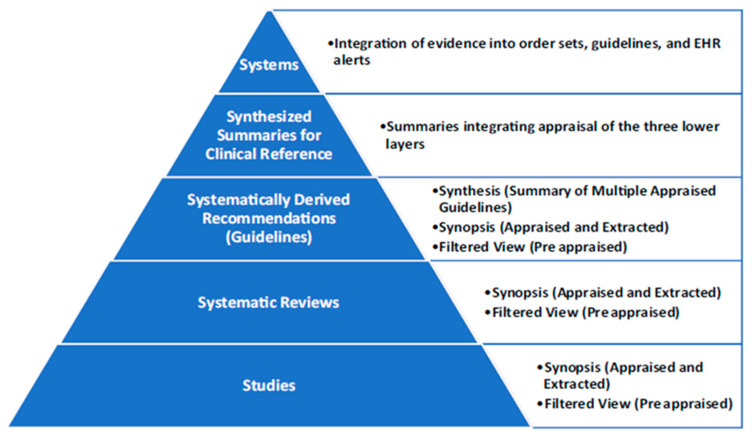
The more recent version 5.0 of the pyramid for EBM decision-making (Adapted from Alper BS, Haynes RB. EBHC pyramid 5.0 for accessing preappraised evidence and guidance. Evid Based Med. 2016;21(4):123–125; with permission) [16].

The hierarchy of medical resources from bottom to top has been recommended by Haynes based on the “5S” pyramid of which was later revised in 1996 [7,8]. An effective search strategy is essential for pediatricians to optimize time and effort. This protocol consists of “Studies”, “Summaries”, “Synopses”, “Syntheses”, “Studies” and “Systems” (Figure 2 and Figure 3) and is usually called the “5S” hierarchy [7,13]. A fundamental principle underlying all pyramids is that evidence with lower quality (higher risk of bias) is positioned at the base of the pyramid, whereas evidence with higher validity occupies the apex. Additionally, the effort and expertise required to utilize the evidence increase ascending the pyramid. Therefore, a search for a clinical answer should ideally start at the apex of the evidence pyramid [14]. Evidence pyramids serve as general guides to the quality of evidence rather than strict rules [14]. The pyramid has undergone multiple revisions, while becoming progressively more complex over time (Figure 2).

The most recent iteration of the pyramid model proposed by Haynes and colleagues acknowledges that not all synopses (concise, structured summaries of clinically important, methodologically sound studies published in evidence-based journals) are equivalent. Consequently, synopses are further categorized into two levels: synopses of individual studies and synopses of syntheses [14]. Alper and Haynes propose an evolution to ‘pyramid 5.0’ consisting of five hierarchical levels: individual studies, systematic reviews, systematically derived recommendations (guidelines) and evidence-based synthesized summaries for clinical reference (such as online textbooks). This structure facilitates detailed patient evaluation, guiding EBM in identifying appropriate interventions to achieve optimal management and favorable disease outcomes [16].

The latest evolution of the pyramid model by Haynes and colleagues is a simplified version of the pyramid created in 2016, comprising five levels that are practical for use in clinical practice (Figure 2). The base of the pyramid contains the largest number of studies, with the strength of evidence increasing at every subsequent level. Ascending to the top, the evidence has been reappraised and translated into guidelines enabling clinicians to readily apply it in their clinical practice [16]. While existing information resources fall short of perfection, currently, there has been considerable progress in providing high-level services in recognizing and applying the most robust information in the topic areas of interest [16,17].

“Studies” occupy the base of the hierarchy; they are time-consuming and require critical appraisal of the retrieved articles. “Summaries” are in the next level of hierarchy, integrating the best available evidence from the lower tiers of medical information to provide a comprehensive overview of management options for a given health problem. “Syntheses” consist of systematic reviews of special medical topics. A systematic review identifies all primary studies relevant to a particular clinical scenario, critically appraises them, and summarizes the findings. Meta-analyses, a subset of systematic reviews, quantitatively combine and analyze results from all relevant studies. While databases such as Medline and SCOPUS index systematic reviews, the most efficient approach to locating these articles is through databases specifically dedicated to systematic reviews. Cochrane Library (www.cochrane.org, accessed on 11 August 2025) represents the gold standard in this regard [17]. “Synopses” include information from original studies and systematic reviews of special medical topics, usually commented on by an expert on the study results. “Systems” occupy the apex of the evidence hierarchy, automatically linking a patient’s specific condition to the relevant clinical information. Comparing the 5S approach with how we usually seek EBM signifies that it is time to revise our everyday clinical practice and adopt an updated approach [18].

## 5. Rating Quality of Evidence and Strength of Recommendations GRADE System (Classification of Quality of Evidence and Strength of Recommendation): Going from Evidence to Recommendations

In the GRADE approach, the composite desirable effects of a management strategy are compared to the composite undesirable effects, and the strength of a recommendation reflects the extent to which we can be confident in [19,20]. Reduction in morbidity and mortality, improvement in quality of life are considered to be desirable effects of an intervention [21]. The GRADE system evaluates the balance of outcomes of interest across alternative management strategies based on four key domains. The effects of desirable and undesirable outcomes, confidence in the effectiveness of the recommendation, assessment of patients’ values and preferences, and resource utilization are all estimated. Furthermore, it encourages physicians, whenever possible, to provide explicit and well-justified suggestions. The strength of a recommendation has important implications for patients, the public, clinicians, and policymakers. In certain cases, guideline writers may issue “only-in-research” recommendations. The GRADE system encourages medical authorities, whenever feasible, to publish explicit and clearly justified recommendations [20]. In the updated GRADE system, recommendations are classified either as strong or weak. The implications of a strong recommendation are [21,22]:

**For patients:** most people in your situation would prefer the recommended course of management, and only a small minority opt against it. The physician should perform a detailed discussion if the patient is reluctant about the treatment or intervention.

**For clinicians**: the majority of patients should receive their recommended course of action.

**For policymakers**: the recommendation can be adopted as a policy in most situations.

In most scenarios, the adopted policy should strive to balance the burden of treatment—such as medication administration or blood testing—while concurrently minimizing the resource expenditures. Adverse outcomes may encompass side effects that negatively influence morbidity, mortality, or quality of life, or that result in increased use of resources.

The implications of a weak recommendation are [21,22]:

**For patients:** most people in your situation would prefer the recommended course of action, but many would choose otherwise.

**For clinicians:** it is important to understand that different patients may require different approaches, and your role is to support each individual in making a management decision that aligns with their personal values and preferences.

**For policymakers:** developing policies will necessitate extensive discussion and engagement with a wide range of stakeholders.

A major constraint in nearly all published articles is the scarcity of high-quality evidence addressing clinical scenarios encountered in current practice. However, authors have provided the current evidence briefly, fitting the clinical points into one concise medical situation, including those from minority groups. We hope that readers will achieve their best within their routine clinical practice to help improve pediatric clinical care and overcome disparities between indigenous and native children, and support effectively the care of children from minorities, Roma, immigrants, and refugees. However, there is a paucity of data in the health management of children from minorities, so data concerning children from middle–high impact societies should be extrapolated and applied to minority children. Of course, in the current multicultural society, research should focus on the special health needs of the indigenous children [22]. Explicit delineation of the evaluative process for the four domains—risk of bias, inconsistency, indirectness, and imprecision—can play an important role in promoting transparency and reliability of medical decisions [20].

## 6. Plan-Do-Study-Act (PDSA) Act and Children—The Most Commonly Used Quality Improvement Tool

Continuous Quality Improvement (CQI) refers to the systematic and ongoing enhancement of processes, patient safety, and quality of care. The objectives of CQI encompass improving operations, clinical outcomes, systems, workflows, workplace environment, and adherence to regulatory standards. Such process improvements may occur incrementally over time or manifest as significant, transformative changes [23]. The model for improvement provides a structured and practical framework for healthcare practitioners to enhance patient outcomes [24]. Effective use of such models requires clinicians to gain familiarity through practice, with both knowledge and competence being essential for developing skills necessary to engage in quality improvement initiatives. As technologies for collecting care-delivery data and methods for tracking outcomes become increasingly sophisticated and integrated into healthcare, continuous quality improvement will play an essential role in ensuring appropriate, efficient, and patient-centered care while supporting healthcare provider satisfaction and prudent resource utilization [25]. An example of objective assessment in decisions in pediatrics is the Plan-Do-Study-Act (PDSA). The Plan-Do-Study-Act (PDSA) methodology is one of the most widely utilized methodologies in quality improvement. The PDSA cycle is an iterative, four-step model for improving a therapeutic process [25,26]. Plan-do-study-act is a guide tool kit provided by the WHO for countries to use rapid-cycle problem-solving to implement family planning guidelines. The World Health Organization (WHO) has also adopted PDSA as a key component of quality improvement initiatives [23,26]. Rapid-cycle problem-solving needs a coordinated team of medical and nursing personnel. A leader will take responsibility for pulling together a team, organizing the process, and following its progression so as to identify a valid solution [23].

The PDSA cycle has 4 steps [25]:

Step 1: **Plan** (observations, collections of data). States the objective of the test, the questions the test will be designed to answer, makes predictions about what the results of the test will be, and develops a plan to test the change.

Step 2: **Do.** This involves implementing the plan by executing the test as designed, documenting any problems or unexpected observations, and initiating preliminary data analysis.

Step 3: **Study.** This involves analyzing the data and studying the results, comparing the data and results to the physician’s predictions, summarizing, and reflecting on what has been learned.

Step 4: **Act.** This entails refining the change based on insights gained from the test, determining modifications, and developing a plan for subsequent testing.

The Plan-Do-Study-Act (PDSA) method is widely employed in quality improvement initiatives. However, prior research has highlighted frequent methodological shortcomings in PDSA-based problems. A recent systematic review of 120 quality improvement initiatives found that nearly all projects (98%) reported some form of improvement [27]. However, only 32 (27%) specified a concrete, quantitative aim and successfully achieved it. Furthermore, 72 projects (60%) provided sufficient documentation of PDSA cycles for comprehensive analysis, and of these, only three (4%) adhered to all four key methodological elements. Even though the majority of projects demonstrated improvement, the pervasive low adherence to methodological standards raises concerns regarding the reliability and justification of PDSA-based quality improvement efforts. This review indicates the ongoing need to strengthen quality improvement in clinical medicine [27].

## 7. The Wisconsin Child Welfare Professional Development System and the HCM—UCLA Healthcare Facility of the Department of Pediatrics of the Public University of California

The Wisconsin Child Welfare Professional Development System is dedicated to providing organizational Process Improvement in children and promoting the PDSA Collaboratives [28]. In a PDSA Collaborative, a team comprising workers and a supervisor(s) is sent by local child welfare agencies to learn how to implement organizational change using the PDSA model. Between planning sessions, these teams engage in action periods during which they test organizational changes within their respective agencies. Although all teams are working on a common change topic, each may experiment with different strategies. Planning sessions, along with technical support, provide opportunities to the teams for joint learning, problem-solving, and sharing resources across participating agencies in the collaborative. The Plan-Do-Study-Act Collaboration focuses on strengthening child welfare and human services/behavioral health to better address the needs of children. Additionally, services for children and families are designed to be family-centered, strength-based, streamlined, supportive, and flexible. Through this process, agencies enhance their understanding of their collaborating partner’s mission and philosophy, establish clear and consistent protocols for coordination, and develop procedures to facilitate information sharing and service access. Ultimately, this approach promotes evidence-based decision-making for clinical care and fosters agreements among collaborating partners to achieve optimal outcomes for families [29].

HCM—UCLA is a healthcare facility of the public University of California, located in both California and Los Angeles, providing a broad assessment of excellence in hospital-based pediatric patient care. The Pediatric Department has created a site which is designed to facilitate access to tools that promote an approach to research tools that facilitate the correct decision-making, patient care, and research based on EBM principles [30] (Figure 1).

## 8. A Model for Improvement in Healthcare Quality in Pediatric Clinical Practice—Continuous Quality Improvement (CQI)

The implementation of CQI is a fundamental requirement for healthcare organizations [24]. CQI philosophy emphasizes a shift from an institutional to an individual focus, obligating each provider to enhance their patients’ outcomes [22]. This approach is grounded in core tenets: organizational commitment to quality, a patient-centric focus, systemic (rather than individual) modification, and collaborative problem-solving, all informed by objective data [24].

However, despite its recognized importance, equated with core medical knowledge, a deficiency in formal CQI training impedes providers’ capacity to execute these quality initiatives [24].

The development of quality measures in both public and private sectors has been influenced by several analytic frameworks. Among the most prominent is the one established by the Institute of Medicine’s 2001 report, Crossing the Quality Chasm, which articulated six fundamental aims for high-quality healthcare [29]:

- **Safe**, by avoiding harm from intended care.
- **Effective**, by utilizing evidence-based science for all who may benefit while preventing both underuse and overuse.
- **Patient-centered**, meaning care is tailored to and guided by individual patient preferences, needs, and values.
- **Timely**, through the reduction in delays.
- **Efficient**, by maximizing resource utilization and eliminating waste.
- **Equitable**, ensuring quality does not vary based on geographic, demographic, or socioeconomic factors.

**Figure 4:**
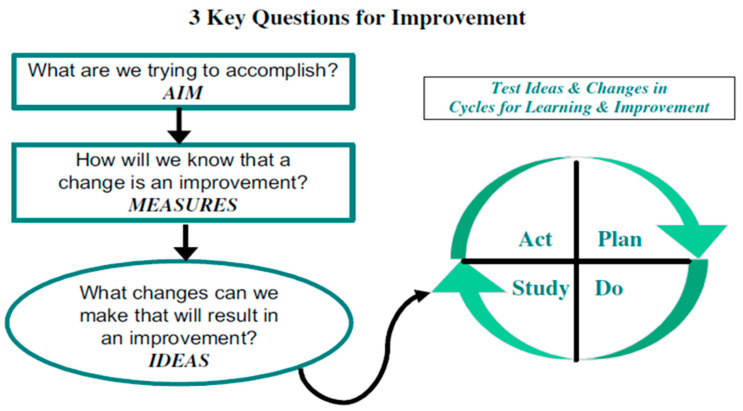
Model of improvement in medical care. Gerald J. Langley, Ronald D. Moen, Kevin M. Nolan, Thomas W. Nolan, Clifford L. Norman, Lloyd P. Provost. The Improvement Guide: A Practical Approach to Enhancing Organizational Performance, 2nd Edition, 2009 (with permission from the publisher John Wiley and sons) [31].

The Model for Improvement is an effective methodology designed to deliver specific, measurable outcomes. Prominent organizations like the Institute for Healthcare Improvement (IHI) utilize this model to drive successful quality improvement initiatives (Figure 4).

The Model for Improvement centers on three significant questions [31]:

1. Define the Aim: “What are we trying to accomplish?” This requires establishing a clear, specific, and agreed-upon goal that is aligned with broader organizational objectives.
2. Establish Measures: “How will we know if a change is an improvement?” This involves selecting relevant metrics (outcome, process, and balancing) that are useful, integrated into daily work, and displayed visually to track progress.
3. Identify Changes: “What changes can we make that will result in improvement?” This step involves brainstorming change ideas from various sources (research, observation, creativity) and selecting the most promising ones based on their potential impact, feasibility, and relevance to the aim [31].

Phase 1—Plan: Prepare or determine the modifications, changes, or alterations that would achieve the aim.

Phase 2—Do: Conduct the test, collect the data, and then provide analysis.

Phase 3—Study: Sequential analysis of data, assuming proposed changes under consideration.

Phase 4—Act: Implement the change or modification of the process.

Seven critical factors determine whether physicians perceive data feedback as an effective tool for performance improvement: (1) the perceived validity of the data is a primary motivator for change; (2) data credibility within an institution develops over time; (3) the source and timeliness of data are fundamental to its acceptance; (4) benchmarking enhances the context and relevance of feedback; (5) physician leaders play a key role in enhancing the effectiveness of data dissemination; (6) powerful, individualized performance profiles may be perceived as punitive; (7) sustained data feedback is necessary to maintain improved performance [32].

### 8.1. A. Creating Systematic Research Strategies

#### Creating a Systematic Research Strategy—The Medical Library Association (MLA)

The Medical Library Association (MLA) is a global, non-profit educational organization, supported by the University of Vermont, which is dedicated to empowering health information professionals. It provides its members with resources for community engagement, funding, continuing education in health information management, and support for research and evidence-based practice. A central part of MLA’s mission is to facilitate collaboration between information professionals and clinical experts. This partnership is essential for equipping clinicians with the modern skills necessary to enhance their practice through EBM. The MLA outlines a specific methodology for planning and creating a comprehensive search strategy across multiple databases, which consists of the following steps [33]:

1. Formulate a focused research question using a structured framework.
2. Define the desired study types and evidence that would best address the question.
3. Extract key concepts from the question to guide search term selection.
4. Select the most critical concepts to balance precision.
5. Identify relevant databases and platforms.
6. Keep a detailed search log.
7. Find controlled vocabulary for each key concept.
8. Collect synonyms and related terms for comprehensive coverage.
9. Include spelling variants and word forms to capture all relevant results.
10. Apply advanced search techniques using Boolean operators, field codes, and truncation.
11. Refine the search strategy by adjusting terms and limits based on initial results.
12. Review retrieved articles for relevance and quantity.
13. Verify the search syntax and correct any errors or oversights.
14. Adapt the search strategy for other databases using their respective vocabularies and syntax.
15. Iteratively test and refine the search across databases.

### 8.2. B. Creating a Systematic Research Strategy-PubMed Central^®^(PMC) and the Cochrane Library

PubMed remains the leading gateway to biomedical and life sciences literature, serving as the cornerstone for more specialized research databases. It has evolved to include PubMed Central^®^ (PMC), a free full-text digital archive maintained by the U.S. National Institutes of Health’s National Library of Medicine (NIH/NLM). Located in Bethesda, Maryland, the NLM is the world’s largest biomedical library. It houses an extensive print collection and develops electronic resources that are accessed billions of times annually by users worldwide. In addition to providing access to scientific literature, the NLM also supports and conducts research, development, and training in the fields of biomedical informatics and health information technology [34]. In accordance with the NLM’s mission to collect and preserve biomedical literature, PMC is a key component of NLM’s holdings. This collection also includes NLM’s extensive print and licensed electronic journals, all of which support modern biomedical and healthcare research. All content in PMC is free to access, reflecting NLM’s commitment to long-term digital preservation through active and widespread use of the archive [35]. It is important to note that while PMC offers free access to its materials, content remains protected by copyright. PMC enables efficient, cross-referenced searches across diverse sources stored in a unified format, allowing users to quickly retrieve full-text articles and locate all relevant information. The archive also facilitates the integration of scientific literature with other data resources. To refine search results and reduce the volume of literature retrieved, users can take advantage of PubMed’s “Limit” section or the “Clinical Queries” feature.

The Cochrane Library serves as a leading global, non-profit collaboration composed of health researchers, clinicians, patients, and caregivers dedicated to generating and disseminating reliable health information. Its mission is to improve health outcomes worldwide by ensuring that health decisions are grounded in up-to-date, credible, and applicable evidence. As an independent organization, Cochrane works with partners across the world to create accessible and trustworthy evidence, while also advocating for its application in promoting equitable and effective healthcare. Using advanced methodological techniques, Cochrane systematically combines and compares, sometimes from hundreds of studies, to produce synthesized results that are more robust than those from any single study. These comprehensive syntheses are known as ‘systematic reviews’. Recognized internationally for methodological rigor, Cochrane systematic reviews are highly regarded by healthcare professionals, researchers, and policymakers as a gold standard in evidence-based health information [36].

**Figure 5:**
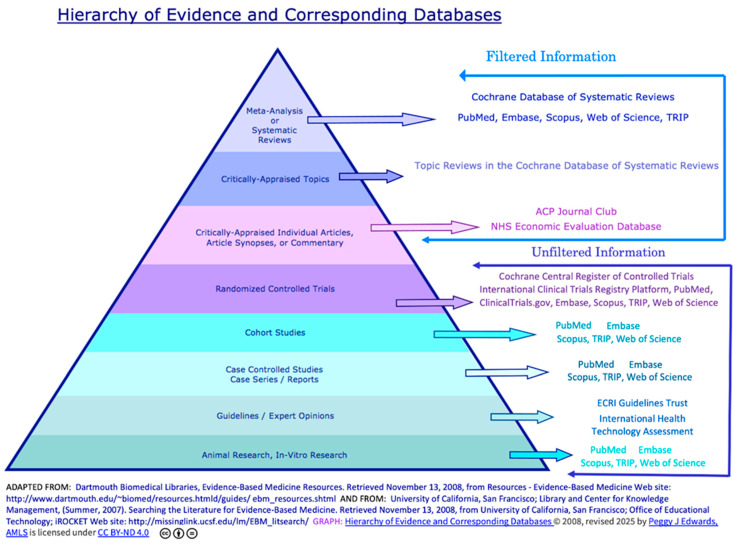
Hierarchy of Evidence with filtered and unfiltered information [38,39]. ADAPTED FROM: Dartmouth Biomedical Libraries, Evidence-Based Medicine Resources. Retrieved 13 November 2008, from Resources-Evidence-Based Medicine Web site: https://home.dartmouth.edu/ (accessed on 11 August 2025) AND FROM: University of California, San Francisco; Library and Center for Knowledge Management, (Summer, 2007). Searching the Literature for Evidence-Based Medicine. Retrieved 13 November 2008, from University of California, San Francisco; Office of Educational Technology; Web site: https://ttuhsc.libguides.com/c.php?g=1467082&p=10914682&preview=d21c35ad203d503248f2211a0eb19176 (accessed on 11 August 2025). GRAPH: Hierarchy of Evidence and Corresponding Databases © 2008, revised 2025 by Peggy J Edwards, AMLS is licensed under CC BY-ND 4.0.

The Cochrane Library is an international, not-for-profit organization headquartered in the UK and a member of the UK National Council for Voluntary Organizations. It produces and publishes systematic reviews freely accessible, allowing anyone seeking reliable, high-quality health information to use this resource for guidance [36]. Cochrane evidence serves as a valuable resource for clinicians, patients, caregivers, researchers, and policymakers alike, supporting informed healthcare decisions and advancing medical knowledge. The Cochrane Library consists of three main sections, including the Cochrane Database of Systematic Reviews (CDSR), which contains full reviews (systematic), review protocols, editorials, and supplements. It addresses a wide range of healthcare topics, including health services, and is developed and maintained by Cochrane. Cochrane review authors are encouraged to regularly update their published reviews to incorporate new evidence as it becomes available. This practice helps maintain clinical relevance and accuracy, ensuring that healthcare providers, patients, and policymakers have access to the most current and reliable information for decision-making. The CDSR is a leading resource for high-quality systematic reviews in healthcare [36]. The Cochrane Database of Abstracts of Reviews of Effects (DARE), produced by the Centre for Reviews and Dissemination (CRD) at the University of York, contains 15,000 abstracts, each including a summary and critical appraisal, and over 6000 quality-assessed reviews. The Cochrane Central Register of Controlled Trials (CENTRAL) offers an extensive collection of randomized controlled trial reports, serving as a valuable source of high-quality evidence (Figure 5).

A cross-sectional audit of reviews published between January 2012 and August 2013 in the CDSR and DARE revealed a moderate correlation between the number of systematic reviews produced and disability-adjusted life years. However, no correlation was found between review output and disease burden measured by mortality rates. The audit also highlighted that certain areas, such as mental and behavioral disorders, musculoskeletal conditions, and other non-communicable diseases, were overrepresented in these databases relative to their contribution to overall mortality [37].

### 8.3. C. Creating a Systematic Research Strategy-Users, Doers, and Replicators

Since the emergence of EBM, the clinical learning environment has transformed significantly. Technological innovations, such as internet access via laptops and mobile devices, have reshaped clinical settings, enabling efficient searches through rapidly expanding medical literature and supporting real-time integration of evidence into patient care. This shift requires healthcare providers to skillfully navigate increasingly complex medical information and apply it appropriately in practice. The choice of resource often depends on the clinical context: a practitioner might use the Cochrane Library or the Trip Database to quickly locate systematic reviews or guidelines during busy clinical duties, while a researcher may turn to PubMed for deeper engagement with primary literature.

Regardless of setting, whether as a consultant in a pediatric hospital or as a trainee, there are multiple ways to incorporate evidence into practice. Straus and McAlister’s model categorizes these approaches through the roles of users, doers, and replicators, offering a framework for understanding how evidence can be accessed, applied, and shared in clinical environments [40]. Most physicians are considered users of evidence, as they rely on pre-appraised resources, such as the Cochrane Library or Trip Database, to inform their clinical decisions. This approach is efficient and well-suited to fast-paced care settings, through its usefulness depends on the availability of existing summaries on relevant topics [41]. In contrast, doers engage deeply with the evidence pyramid, conducting comprehensive literature searches, critically appraising studies, and applying full EBM processes before making clinical decisions. While this method is more time-intensive, it is especially valuable for addressing common or significant clinical questions where robust evidence is needed [41]. A third approach involves replicators, clinicians who adopt recommendations from local opinion leaders without independently reviewing the underlying evidence [41]. This strategy can be useful in complex, rare, or unusual cases where expert guidance is beneficial. However, it carries the risk of uncertainty regarding how much evidence actually informs the expert’s opinion.

A practical way to begin practicing evidence-based medicine is to learn from experienced clinicians who already apply evidence in their practice [42]. In a Canadian survey of internists, respondents believed that adopting evidence-based guidelines was the most effective approach to practicing evidence-based care [43]. To easily incorporate EBM into your daily routine, it is recommended that you become a proficient “user” of evidence by applying the following strategy [41]:

1. Select just two trusted evidence-based clinical resources.
2. Ensure these resources are easily accessible during patient care.
3. Focus first on common or high-impact conditions you encounter regularly.
4. Combine the evidence you find with your own clinical expertise and your patients’ values and preferences.

## 9. “Archimedes” and the AGREE II Checklist

To deliver the best possible care to patients and their families, pediatricians must combine high-quality scientific evidence with their own clinical expertise and the preferences of the family [44].“Archimedes” is a feature within the Archives of Disease in Childhood (published by the BMJ) designed to support clinicians by offering evidence-based answers to common, yet often overlooked, clinical questions in Pediatrics. Launched in September 2001, this bimonthly section summarizes and appraises available evidence to aid everyday decision-making [44].

“Archimedes” also invites pediatricians to contribute their own evidence summaries, particularly those grounded in existing systematic reviews. Readers interested in exploring clinical questions are encouraged to review previously answered topics. If their question remains unaddressed, they may submit a summary following the journal’s guidelines available at www.archdischild.com (the Archives of Disease in Childhood) (accessed on 11 August 2025). This issue of the journal covers three such topics [45,46,47].

When evaluating the effectiveness of diagnostic tests, researchers must compare the test’s ability to detect or rule out a condition against an established reference standard. This evaluation should be conducted in a population similar to the one in which the test will ultimately be used. Among those who test positive, some will truly have the condition (true positives), while others will not (false positives). Similarly, some who test negative will truly not have the condition (true negatives), while others may actually have it (false negatives).

To assess the trustworthiness of clinical guidelines, pediatricians and other clinicians can use the AGREE II (Appraisal of Guidelines for REsearch and Evaluation) checklist. Rather than asking “Can I use this guideline?”, AGREE II helps answer “Can I trust this guideline?”. It evaluates how guidelines were developed, including how evidence was gathered, assessed for bias, and integrated with clinical expertise and patient values, and whether recommendations are free from undue influence. The AGREE II offers a systematic and validated method for appraising the quality and transparency of clinical practice guidelines (CPGs). It examines six domains: scope and purpose, stakeholder involvement, rigor of development, clarity of presentation, applicability, and editorial independence. The tool includes 23 specific criteria and is widely recognized for its reliability in supporting evidence-informed practice [48]. Clinicians can access tools, training, and support for using AGREE II through the AGREE Enterprise website: www.agreetrust.org (accessed on 11 August 2025) [48].

A recent study on clinical practice guidelines (CPGs) for managing fever symptoms in children found that GRADE II guidelines were suitable for use, though some modifications were recommended, particularly in the areas of methodology, applicability, and editorial independence. These findings may help enhance the development of future guidelines and support more informed selection and application of CPGs in clinical practice [49].

## 10. Good Clinical Practice Principles for Clinical Research in Children and Adolescents

Good clinical practice is an internationally recognized ethical and scientific standard governing the design, conduct, monitoring, recording, analysis, and reporting of clinical trials involving human participants. It ensures that trial results are reliable and accurate, and that the rights, safety, confidentiality, and well-being of participants are protected [50].

The ethical foundation for human research was significantly advanced in 1964, when the World Medical Association adopted the Declaration of Helsinki during its 18th General Assembly. This document outlines essential ethical principles for medical research involving human subjects and has undergone multiple revisions to remain relevant to evolving research contexts [51].

GCP is not only an ethical guideline but also a legal requirement in health systems worldwide. Regulatory authorities will not approve new medical products or interventions unless they have been developed in compliance with GCP standards [52].

The United Nations Convention on the Rights of the Child (UNCRC), established in 1989, legally affirms that every child, irrespective of race, religion, or ability, is entitled to civil, political, economic, social, and cultural rights. It states that “the best interests of the child shall be a primary consideration” in all decisions affecting them, and recognizes each child’s right to “the highest attainable standard of health” [53]. Because children and adolescents constitute a vulnerable population, clinical trials involving them require special ethical and operational considerations. These trials must be carefully designed and conducted according to GCP and high ethical standards, with particular attention to developmental differences between pediatric and adult populations. Such research is essential to generating evidence for safe, effective, and age-appropriate medical treatments [54].

The World Health Organization’s International Clinical Trials Registry Platform (ICTRP) strives to make clinical research more transparent and accessible. It supports ethical and relevant pediatric trials by improving access to guidelines, regulations, and trial data, thereby helping ensure that clinical research benefits all children [54].

Key ethical requirements in pediatric trials include obtaining written informed consent from parents or guardians and, where appropriate, seeking assent from the child participants. Institutional ethics committees must review and approve all trials to safeguard participants’ rights and welfare, and ongoing monitoring is necessary to ensure data quality and participant safety [55].

Researchers conducting pediatric trials must be specially trained, committed to pediatric health needs, and work within adequately resourced settings. Facilities, equipment, data confidentiality, and product storage must meet rigorous standards. Above all, pediatricians and investigators must prioritize children’s health and well-being throughout the research process [55,56,57].

The prerequisites for conducting a clinical trial in children in accordance with Good Clinical Practice involve [55]:

1. Selection of trial subjects (Target population).
2. Protection of trial subjects (children represent a vulnerable population).
3. Safety reporting (monitor and record side-effects).
4. Develop simple and lay language for ensuring understanding of informed consent forms by the children’s caregivers.
5. Ethical approval by the Institutional Review Board (IRB) or Research Ethics Board (REB).
6. High quality of data.
7. Explain resources (funding, equipment, an adequate number of children available for recruitment).
8. Randomization (preferably by an electronic system).
9. Documentation and reporting (accurate reporting, interpretation, monitoring, and verification of the quality of all aspects of the trial).
10. Monitoring in pediatric clinical trials ensures the quality of medical care, data accuracy, and trial integrity, while safeguarding the health and rights of enrolled children. It involves preventing, detecting, and addressing errors, negligence, fraud, misconduct, or protocol violations.
11. Monitor visits.
  (a) Pre-trial monitoring visit: The principal investigator educates the research team, assesses trial feasibility at the site, and confirms the commitment of the investigators.
  (b) Trial initiation visit: The study coordinator provides detailed information to the research team, distributes study materials and documents, and ensures the team understands the trial protocol and GGP requirements.
  (c) Ongoing monitoring visits: Patient progress and outcomes are tracked, and data collection is verified for accuracy and compliance.
  (d) Close-out visit: The study coordinator ensures proper archiving of the investigator’s files and collects all unused materials, documents, or products.
12. Compliance of the investigator with his/her responsibilities after approvals and during the study.

## 11. Discussion

Since its inception three decades ago, EBM has become an integral component of clinical medicine. The five-step process (ask, acquire, appraise, apply, assess) continues to provide a valuable framework for articulating EBM’s principles, developing curricula, and evaluating performance at individual and institutional levels. It is critical to acknowledge that EBM is implemented within a complex ecosystem. Practitioners must assume multiple roles to navigate a multitude of external evidence sources while also contending with influences from regulatory bodies and payers. The ultimate aim of EBM, determining if evidence is applicable to an individual patient, is a multifaceted process involving ethical, socio-economic, and personal considerations.

Practicing EBM requires mastering challenging new skills in the literature search and critical appraisal. Despite this hurdle, many clinicians are interested in developing these competencies [58,59]. EBM faces several practical challenges, including difficulty in low-resource settings, a lack of high-quality evidence for all questions, and the complexity of applying generalized evidence to individual patient needs. Time constraints and limited proof of EBM’s efficacy on outcomes are also noted [40]. Common misconceptions are that EBM undervalues clinical expertise, ignores patient preferences, and promotes “cookbook” medicine. In reality, it integrates expertise and patient values to inform, not replace, clinical judgment. While EBM is resource-intensive, future efforts should focus on improving evidence access, enhancing communication for shared decision-making, and researching its impact on patient outcomes [40].

Another significant concern is the under-representation of patient-oriented outcomes for marginalized groups (minorities, refugees, immigrants, Roma children) in public research. The task of remaining abreast of the exponentially growing body of evidence is itself a formidable challenge. Consequently, the Model for Improvement is proposed as a viable and systematic methodology for healthcare practitioners to improve patient outcomes. Achieving proficiency in this model is essential for effective participation in quality improvement efforts.

Advancing healthcare requires all practitioners to deliver safe, effective, and efficient patient care. The described methodology is readily applicable even in high-demand healthcare environments. By embedding this quality improvement approach into routine practice, clinicians can improve advanced care delivery and system design, thereby enhancing clinical outcomes. Additional implementation support is available through various organizations devoted to healthcare quality improvement.

Regarding the PICO model, there are not enough studies to prove the superiority of using this model versus other available models or unguided searching tools. Therefore, no definite conclusions can be drawn regarding the effect of the model on the quality of the literature search. Well-designed studies are needed to assess the role of structured tools in organizing search strategies before implications for current practice can be established [10,12].

Furthermore, the advent of artificial intelligence and technological innovation in medicine dictates that a substantive evolution of EBM is required, moving beyond simple adaptation. This evolution must be characterized by a merger of EBM’s principles with the objectives of personalized medicine. This synthesis will be facilitated by methodological advances and the application of AI-based analytics to large-scale data, forging a path toward truly individualized care—ensuring the correct therapeutic intervention for the appropriate patient at the optimal time [60].

The medical community anticipates the findings of current rigorous research and hopes they will yield robust conclusions. In the interim, practitioners must rely on extrapolating from the best available evidence. This process necessitates critical evaluation of how an individual patient may differ from research subjects, an understanding of patient-specific priorities, and an analysis of the potential origins of clinical benefit. Crucially, this also entails openly communicating the boundaries of our medical certainty to families.

## 12. Conclusions

EBM is imperfect and is subject to criticism. These criticisms remain somewhat valid, depending on each clinician’s perspective. We must acknowledge EBM’s limitations, which, if applied thoughtfully, serve as a valuable guide in medical decision-making. The advancement of EBM in Pediatrics necessitates a strategic shift beyond mere evidence generation towards a holistic emphasis on the creation of high-quality, clinically relevant evidence and its subsequent accessibility and implementation at the point of care. Realizing this objective mandates a coordinated, multistakeholder initiative across the healthcare continuum. Technology will serve as a fundamental catalyst in this endeavor. By adopting these forward-looking recommendations, the pediatric community can ensure that clinical decision-making is consistently guided by the most robust evidence available, thereby optimizing health outcomes tailored to the distinct physiological and developmental needs of pediatric patients. The tools presented in this review will aid pediatricians in making clinical choices grounded in the strongest, most current, and relevant research. Methods like structured testing can foster innovation, refine treatments, sustain progress, and systematize protocols to assure consistency. Additional support, education, and resource allocation are available through organizations committed to enhancing Pediatric healthcare quality.

## Figures and Tables

**Table 1:** 5 Key steps in patients’ history taking and clinical evaluation essential for implementing ΕΒΜ in Pediatrics.

Step 1	Defining a clinically relevant question
Step 2	Searching for the best evidence
Step 3	Critical appraisal of the found literature/evidence
Step 4	Applying evidence in daily practice
Step 5	Evaluating the performance of EBM

## Data Availability

The original contributions presented in this study are included in the article. Further inquiries can be directed to the corresponding author.

## Abbreviations

The following abbreviations are used in this manuscript:

AGREE	Appraisal of Guidelines, REsearch and Evaluation
BMJ	British Medical Journal
CDSR	Cochrane Database of Systematic Reviews
CPG	Clinical Practice Guidelines
CQI	Continuous quality improvement
CRD	Centre for Reviews and Dissemination
DARE	Cochrane Database of Abstracts of Reviews of Effects
EBM	Evidenced-Based Medicine
GCP	Good Clinical Practice
HCM	Hypertrophic cardiomyopathy
ICTRP	The International Clinical Trials Registry Platform
IHI	Institute of Healthcare Improvement
IRB	Institutional Review Board
MLA	Medical Library Association
NCBI	National Center for Biotechnology Information
NIH	National Institutes of Health
NLM	National Library of Medicine
PDSA	Plan Do Study Act
PICO	Patients’ Intervention Comparison Outcome
PMC	PubMed Central
REB	Research Ethics Board
U.S.	United States
UCLA	University of California, Los Angeles
UK	United Kingdom
UNCRC	United Nations Convention on the Rights of the Child
WHO	World Health Organization
